# Upregulation of *HSF1* in estrogen receptor positive breast cancer

**DOI:** 10.18632/oncotarget.12438

**Published:** 2016-10-04

**Authors:** Yesim Gökmen-Polar, Sunil Badve

**Affiliations:** ^1^ Departments of Pathology and Laboratory Medicine, Indianapolis, IN; ^2^ Department of Medicine, Indiana University School of Medicine, Indianapolis, IN; ^3^ Departments of Indiana University Melvin and Bren Simon Cancer Center, Indianapolis, IN

**Keywords:** Breast cancer, HSF1, genomics, estrogen receptor, endocrine resistance

## Abstract

Heat shock transcription factor 1 (*HSF1*), a key regulator of the heat-shock response, is deregulated in many cancers. *HSF1* can mediate cancer cell survival and metastasis. High levels of *HSF1* have been associated with poor prognosis in breast cancer. The nature of *HSF1* upregulation needs to be validated in different cohorts to further validate its prognostic utility in breast cancer.

We first evaluated its expression in a cohort of breast cancer tissue microarrays with Onco*type* DX recurrence scores available using immunohistochemistry. To further confirm the clinical relevance and prognostic impact, mutational and methylation status of the gene were also assessed in The Cancer Genome Atlas and publically available microarray datasets.

Immunohistochemical analysis showed that HSF1 expression is independent of Onco*type* DX high recurrence score in ER-positive node-negative patients. Analysis of The Cancer Genome Atlas data revealed upregulation of *HSF1* is not due to methylation or mutation. *HSF1* copy number variations and amplifications (15%) were not associated with survival. In publicly available microarray datasets, a prognostic impact was observed in ER-positive tumors, but not in ER-negative tumors. Patients with ER-positive tumors with high *HSF1* levels were associated with shorter overall survival (*P* = 0.00045) and relapse-free survival (*P* = 0.0057). In multivariable analysis, *HSF1* remained a significant prognostic parameter.

The mRNA expression levels of *HSF1* in ER-positive breast cancer are associated with both shorter relapse-free and overall survival. This prognostic impact is specific to mRNA expression, but stayed insignificant by protein expression or by analyzing amplification events.

## INTRODUCTION

Cells and organisms respond to stress by inducing heat shock proteins which act as molecular chaperones to restore protein homeostasis [1–4]. This adaptive mechanism is controlled by heat shock transcription factor (*HSF1)*. When this transcription factor is activated, it gets phosphorylated, trimerized, and translocates to the nucleus. In the nucleus, it binds to specific DNA sequence motifs (known as heat shock elements) leading to the synthesis of heat shock proteins. In most experimental models, *HSF1* enables adaptive changes in a diverse array of cellular processes including signal transduction, glucose metabolism and protein translation [5–10]. The binding of HSF1 to the DNA is dramatically different based on the phase of the cell cycle. HSF1 binds to only 35 target sites in mitotic chromatin, as opposed to 1242 target sites in freely cycling cells [11]. Its ability to activate transcription in mitosis is minimal. Consequently, mitotic cells are unable to induce expression of heat shock genes and are susceptible to protein damaging stress.

Cancer cells, being mutation prone and aneuploid, show a high activity of *HSF1*. Recently, Mendillo et al. identified genomewide target sites of *HSF1* in breast cancer cell lines with different metastatic capacities [12]. They showed that *HSF1* driven transcription is profoundly different in malignant cells compared with cells that are exposed to heat stress. Cancer cells seem to “hijack” *HSF1* and utilize its transcriptional activity and central role in homeostasis to promote their growth and metastatic potential [13]. The importance of *HSF1* in carcinogenesis is demonstrated by the dramatic reduction in susceptibility of *HSF1*-knockout mice to a wide spectrum of carcinogens [5, 14]. Similarly, depletion of *HSF1* leads to marked decrease in proliferation and survival in established human cancer of cell lines [5, 9, 10, 14].

The role of *HSF1* in breast cancer is not well established. Xi et al. have documented that deletion of *HSF1* in mice overexpressing ERBB2 significantly reduces mammary tumorigenesis [15]. In addition the mice show a significant reduction in lung metastasis. Santagata et al. analyzed the expression of HSF1 in breast cancer samples from the Nurses' Health Study using immunohistochemistry [16]. They have documented an association of high HSF1 expression with increased mortality particularly in ER-positive patients (HR 2.1; *P* < 0.0001). They have postulated that targeting *HSF1* might be a useful therapeutic strategy. In contrast Cheng et al. did not find *HSF1* to be important in multivariate analysis in breast cancer [17]. In the current study, we focus on validating these findings using data from publicly available gene expression databases, including The Cancer Genome Atlas (TCGA), as well as by performing immunohistochemistry using a commercially available antibody. We confirmed that high expression of *HSF1* mRNA, but not amplification, is associated with poor prognosis. However, we found only a weak association between protein expression and high Onco*type* DX recurrence scores, a surrogate for adverse outcomes.

## RESULTS

### Upregulation HSF1 protein levels is independent of Onco*type* DX high recurrence scores

**Figure 1:**
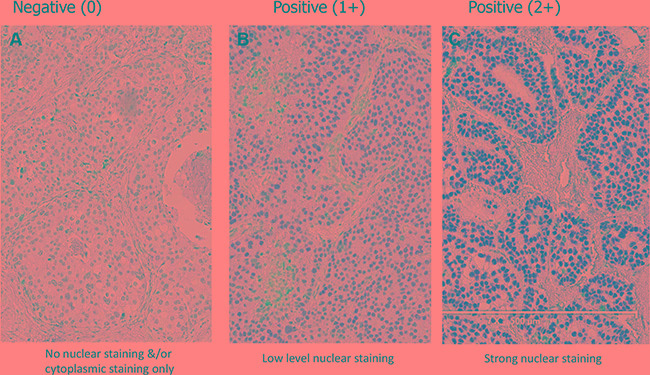
Expression of *HSF1* in breast cancer Representative immunohistochemical staining pattern of primary breast tumors for *HSF1* (New England BioLabs, #4356). Nuclear staining intensity is categorized as the following; (**A**) no nuclear staining &/or cytoplasmic staining only (0), (**B**) low level nuclear staining (1+), (**C**) strong nuclear staining (2+).

Analysis of the National Surgical Adjuvant Breast and Bowel Project (B14 and B20) clinical trials has led to the development of the Onco*type* DX recurrence score [20]. This score estimates the likelihood of disease recurrence in women with early-stage, ER-positive breast cancer and has been used as a surrogate for predicting outcomes. To validate the prognostic relevance of HSF1 observed by Santagata et al, we assessed the expression levels of HSF1 in a TMA cohort of patients with Onco*type* DX scores using a commercially available antibody (see Materials & Methods) [16]. Immunohistochemistry results for HSF1 expression (Figure 1) were assessable for 161 (77.6% of 210) patients (87 low; 54 intermediate and 20 high Onco*type* DX scores). As shown in Table 1, there was no association between Onco*type* DX score and staining intensity (*P* = 0.23), percentage (less or more 10%; *P* = 0.17), or H-score (above or below 70; *P* = 0.08).

**Table 1:** Correlation of the immunohistochemistry findings with Oncotype Dx scores

Table 1	Nuclear Intensity			Percentage positivity		H-Score	
Oncotype DX score	0	1	2	< 10%	> 10%	H < 70	H > 70
High	4	9	7	4	16	9	11
Intermediate	17	22	15	18	36	27	27
Low	36	36	15	38	49	57	30
Grand Total	57	67	37	60	101	93	68

### *HSF1* expression and copy number alterations in primary breast tumors

**Figure 2:**
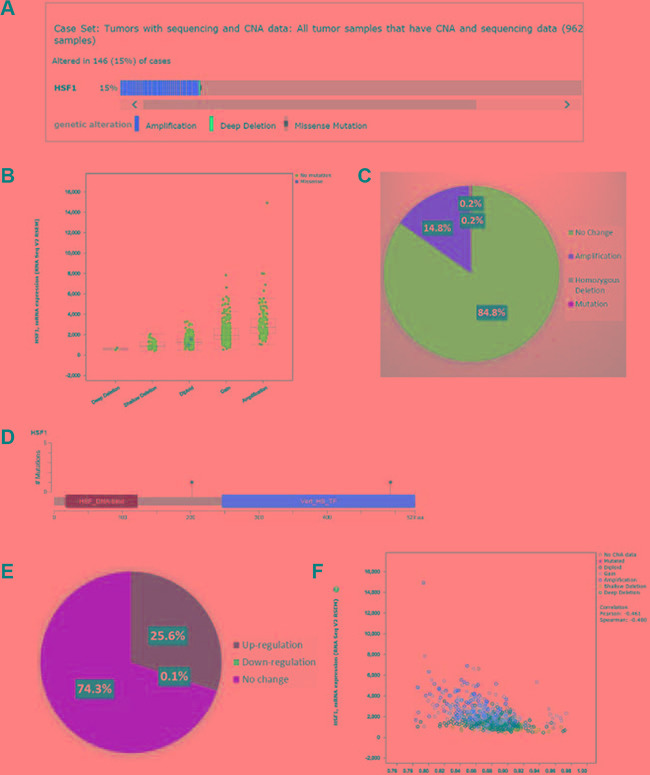
The cBio cancer genomics portal analysis Copy number alterations (CNAs) and Mutations of HSF1 in TCGA breast cancer dataset. (**A**) individual genes are represented as rows, individual cases or patients are represented as columns, and glyphs and/ or color-coding is used to compactly summarize distinct genomic alterations, including somatic mutations and copy number alterations (CNAs), (**B**) correlation of CNAs with *HSF1* expression (RNA-seq), (**C**) the pie chart showing the frequency of genomic alterations and mutations, (**D**) mutation details for *HSF1*, (**E**) the pie chart showing the frequency of upregulated or downregulated gene expression, (**F**) correlation of methylation status (HM450) with mRNA expression (RNA-seq) of *HSF1*.

We next analyzed the genomic alterations in TCGA breast cancer dataset (cBio Cancer Genomics Portal). Copy number alterations (CNA) of *HSF1* were observed in 146 (15%) out of 962 breast tumors (Figure 2A–2C). Of these, 14.8% were due to amplification and 0.2% due to homozygous deletion. In addition, only 0.2% patients had mutations which were not located in major domains (Figure 2D). Of the tumors with altered *HSF1* expression, upregulation (25.6%) was more prevalent rather than downregulation (0.1%) (Figure 2E). These analyses confirm that amplification is the most common alteration, while mutations of *HSF1* are not frequent in breast cancer. Survival analysis of cases with and without amplifications, in all 962 patients or ER+ and ER- subsets did not show an association with overall survival or disease-free survival in TCGA dataset (Supplementary Figure S1). We further analyzed 737 out of 1079 cases available with methylation data (HM450). Methylation was inversely associated with the expression of *HSF1* but the correlation was weak (Spearman 0.45; Figure 2F). However, cases with alterations versus without alterations did not significantly correlate with the overall survival or disease-free survival in TCGA dataset (Supplementary Figure S1).

### High expression of *HSF1* correlates with poor prognosis in patients with ER-positive breast cancer – The Affymetrix microarray datasets

**Figure 3:**
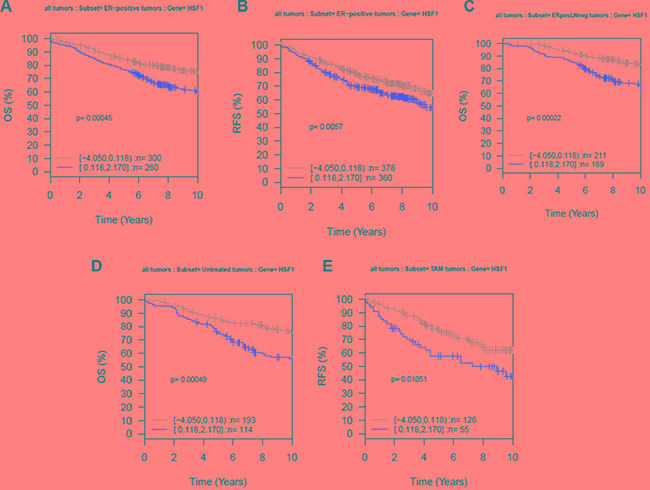
Kaplan-Meier analysis using the Affymetrix data sets (GOBO) Ten-year (**A**) overall survival (OS) and (**B**) relapse-free survival (RFS) for ER-positive tumors, (**C**) ER-positive and lymph node (LN)-negative tumors, (**D**) OS for untreated tumors, and (**E**) RFS for tamoxifen (Tam)-treated tumors. The expression analysis was stratified into two quantiles based on *HSF1* expression; low expression (gray) and high expression (red).

The current study differed from the Santagata study in that a monoclonal antibody was used for the immunohistochemical analysis [16]. To further analyze whether the observed differences could be related to technical issues or biologic relevance, we next evaluated the prognostic value of *HSF1* mRNA expression using overall survival and relapse-free survival as endpoints in subsets of breast cancer represented in 11 microarray datasets (GOBO tool). High expression of *HSF1* was significantly associated with shorter overall survival (ANOVA; *P* = 0.00045), and relapse-free survival (ANOVA; *P* = 0.0057) in ER-positive breast cancer patients (Figure 3A, 3B), but not in ER-negative tumors (data not shown). In this patient group, high expression levels of *HSF1* also correlated with worse overall survival in node-negative subset (ANOVA; *P* = 0.00022; Figure 3C), showing that cases with high *HSF1* have a significant mortality risk when compared with low level of *HSF1* cases. We also assessed the prognostic value of *HSF1* patients in untreated versus tamoxifen-treated patients. High *HSF1* expression is associated with shorter overall survival (ANOVA; *P* = 0.00049) in untreated population (Figure 3D) and with shorter relapse-free survival for tamoxifen-treated patients (Figure 3E). These results indicate that targeting *HSF1* may help prevent development of metastases in ER-positive patients.

Multivariable Cox hazard analysis of *HSF1* expression with clinical variables showed that low *HSF1* expression was associated with good prognosis in all patients (overall survival (hazard ratio [HR] = 0.65; 95% CI = 0.47–0.89; *P* = 0.0083) and relapse-free survival (HR = 0.71; 95% CI = 0.51–1; *P* = 0.048) (Table 2). The other factors that retained significance in multivariable analysis were nodal status, tumor grade, age and tumor size (> 2 cm) for the overall survival. Tumor size (> 20 mm) was the only significant factor for relapse-free survival (HR = 1.98; 95% CI = 1.5–2.62; *P* = 1.58e–06). These results further validate high *HSF1* is an independent poor prognostic marker in ER-positive tumors using large cohorts of breast cancer patients.

**Table 2:** Multivariable analysis in ER+ patients for overall survival and relapse-free survival (Affymetrix datasets)

	OS		RFS	
Variable	HR (95% CI)	*P* value^*^	HR (95% CI)	*P* value^*^
low HSF1 versus high HSF1	0.65 (0.47–0.89)	0.0083	0.71 (0.51–1)	0.048
LN- versus LN+	0.48 (0.35–0.67)	1.93e-05	0.77 (0.57–1.03)	0.079
Grade 3 versus 1 + 2	1.5 (1.05–2.15)	0.025	1.25 (0.92–1.7)	0.148
Age > 50yrs versus < 50yrs	1.49 (1.05–2.13)	0.025	0.82 (0.62–1.1)	0.18
Tumor size > 20 mm versus < 20 mm	2.07 (1.48–2.9)	2.54e-05	1.98 (1.5–2.62)	1.58e–06

## DISCUSSION

Santagata et al, in their study of the Nurses Health Cohort, demonstrated that *HSF1* is an independent prognostic marker in breast cancer in a TMA-based analysis using immunohistochemical methods [16]. However, their study was performed using a cocktail of three different antibodies to detect *HSF1* protein in TMAs. More recently, a monoclonal antibody directed against *HSF1* has become commercially available. The differences in isotope specificity of this antibody and the antibody-cocktail to HSF1 is not known. Apart from the differences in reagents, our study employed a surrogate endpoint, Onco*type* DX assay, for analyzing prognostic impact. In our study, upregulation of HSF1 protein was independent of Oncotype DX scores. In order to confirm that differences observed could be attributed to technical and tissue sampling issues and not related to biological relevance, we further evaluated the role of *HSF1* in breast cancer using publically available datasets.

Analysis of the TCGA dataset showed that mutations were not common (0.2%); copy number alterations were noted in 14.8%, nearly 50% of which were amplification events. *HSF1* amplification or upregulation of *HSF1* was not associated with prognosis in all patients, or in ER-positive or ER-negative subsets. However, it is recognized that the TCGA dataset has limited followup/survival information, so we further analyzed the prognostic relevance of *HSF1* in breast cancer in a publically available large (*n* = 1881) Affymetrix-based gene expression dataset. These analyses showed upregulation of HSF1 was associated with shorter overall survival and relapse-free survival in ER-positive, but not in ER-negative patients. High*HSF1* expression was significantly associated with worse outcome in treatment-naïve tumors and in node-negative tumors. Cheng et al. [17] have also analyzed the role of HSP90, and HSF1 in multiple data sets including many that are included in the current study. They failed to observe a significant impact of HSF1 in their analysis. The major differences in the two studies are the number of patients analyzed and analytical methods. They focused on subsets of patients with highest (top 10% or 25%) and lowest expression and did not find an impact once clinical confounding features were included. In contrast, our multivariable analysis that included age, tumor grade ER status, and treatment type confirmed that high *HSF1* is an independent factor of poor clinical outcome in ER-positive breast cancer. Collectively, this data makes a strong case for a role of *HSF1* in endocrine resistance and recurrence/metastasis.

*HSF1* is a key transcription factor in the regulation of cellular homeostasis and modulates protein folding, stability, and protein-protein interactions. In response to a variety of stresses, *HSF1* binds to the promoter regions of heat shock protein genes and drives transcription of these genes including *HSP90* and *HSP70*. Although *HSF1* supports the survival of normal cells under stress, aberrant upregulation of *HSF1* has been shown to promote tumor cell survival and cancer progression. Therefore, HSP90 inhibitors can serve as potential target for therapeutics for patients with high *HSF1* in ER-positive breast cancer. However, *HSF1* can serve as a hub regulating a transcriptional program that can be distinct from heat shock protein network [12]. Therefore, better understanding of this program is necessary to target *HSF1* therapeutically using agents other than HSP90 inhibitors.

In summary, high expression of *HSF1* mRNA was associated with both shorter relapse-free survival and overall survival in patients with ER-positive breast cancer. Identification of high *HSF1* could stratify patients at greater risk for recurrence/metastasis development independently of Oncotype DX. Therapies that can downregulate *HSF1* need to be explored to prevent recurrence/metastasis development in ER-positive breast cancer.

## MATERIALS AND METHODS

### Tissue microarray patients

All protocols were reviewed and approved by the Institutional Review Board of Indiana University. All archival formalin-fixed, paraffin-embedded tumor blocks in this study were from patients with ER-positive (greater than 1% expression as per ASCO-CAP guidelines) breast carcinomas at the Indiana University Health Pathology Lab (IUHPL). The tissue samples consisted of a tissue microarray (TMA) containing duplicate (1mm) cores. On all of these 210 tumors, Onco*type* DX recurrence score was available. The distribution of the Onco*type* DX scores in the TMA series was 120 low; 65 intermediate; and 25 high scores (Supplementary Table S1).

### Immunohistochemistry

Archival formalin-fixed, paraffin-embedded TMA blocks were cut at 4μ thickness. After deparaffinization and hydration, sections were incubated with the primary antibody. The HSF1 rabbit polyclonal antibody (New England BioLabs #4356) was diluted to 1/100 stained using the BOND-III (Leica Biosystems, Melbourne) automated stainer with Bond Polymer Refine Detection system (Leica Biosystems Newcastle, DS9800), Bond Diluent (Leica Biosystems Newcastle, AR9352) and ER2 reagent (Leica Biosystems Newcastle, AR9640). The ER2 solution was incubated for 20 minutes followed by a 15minute incubation with the diluted antibody. HSF1 scoring was based on H-Score method combining percentage and intensity.

### Analysis of the cancer genome atlas (TCGA)

Somatic mutation rate, DNA copy number alterations (CNAs), mRNA, and methylation status for *HSF1* were analyzed using the cBioPortal for Cancer Genomics (http://cbioportal.org). The portal is a Web resource to analyze complex cancer genomics data including genetic, epigenetic, gene expression and proteomic events [17, 18]. Tumors with CNA and RNA-sequencing data available were analyzed. For the survival analysis, cases with and without amplifications were identified and their survival data downloaded. Log-rank analysis was performed using Kaplan Meier method using the R statistical package.

### Analysis of publicly available datasets

Expression of *HSF1* was analyzed based on ER status, molecular subtypes, and other clinicopathological parameters using the datasets from the gene expression-based outcome for breast cancer online algorithm (GOBO) [19]. GOBO is a web-based analysis tool that utilizes 11 publicly available Affymetrix U133A gene expression data curated from 1881 breast cancer patients with associated stage, grade, nodal status, and intrinsic molecular classification [19]. Of all 1881 tumors, the groups were distributed as follows: a) ER-positive patients (*n* = 1225) of which 326 patients treated with tamoxifen alone while the remainder 927 did not receive systemic therapy and b) ER-negative patients (*n* = 395). Clinical characteristics of individual datasets were described previously [19]. Association of outcome was stratified into the two quantiles based on *HSF1* gene expression level for each patient cohort with overall survival or relapse-free survival as endpoints and 10-year censoring in the above groups. The Kaplan-Meier survival analysis was calculated using Cox proportional hazard model, and the score test of the proportional hazard model was equivalent to the log-rank test.

## SUPPLEMENTARY MATERIALS FIGURES AND TABLES




