# Supplementary material for: Upregulation of HSF1 in estrogen receptor positive breast cancer
Source: Oncotarget. 2016 Oct 4;7(51):84239–45. doi: 10.18632/oncotarget.12438 (PMC5356658; doi:10.18632/oncotarget.12438)
Supplement: Supplementary file 1 [file oncotarget-07-84239-s001.pdf]

Upregulation of *HSF1* in estrogen receptor positive breast cancer

Supplementary Materials

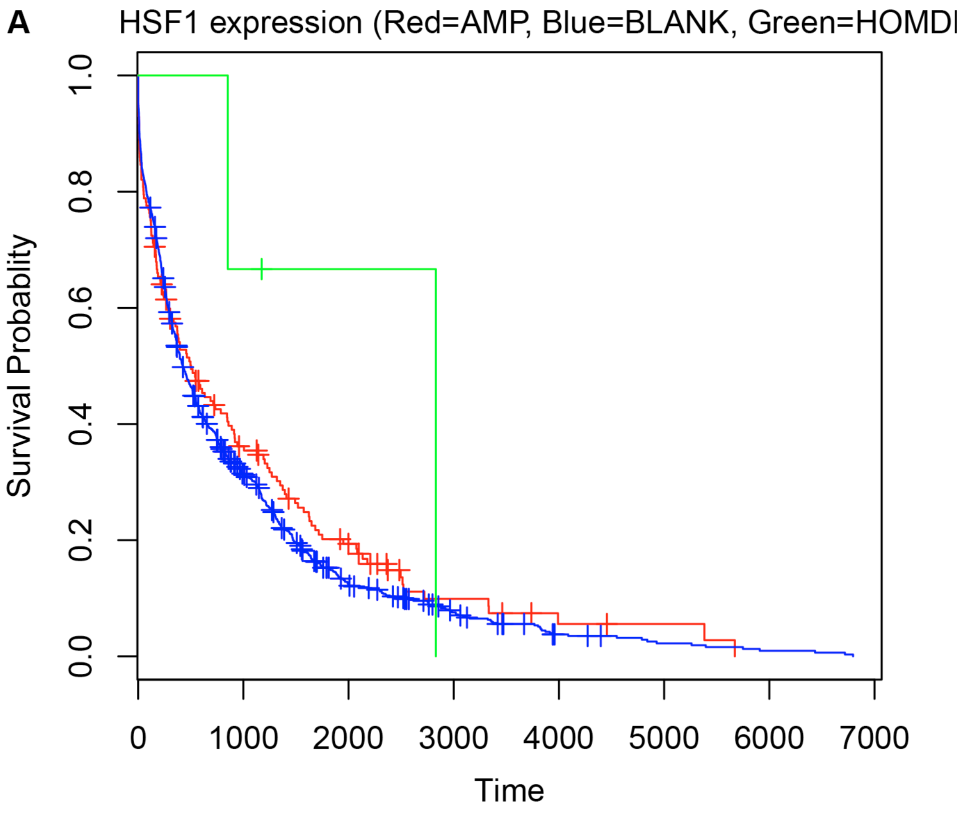

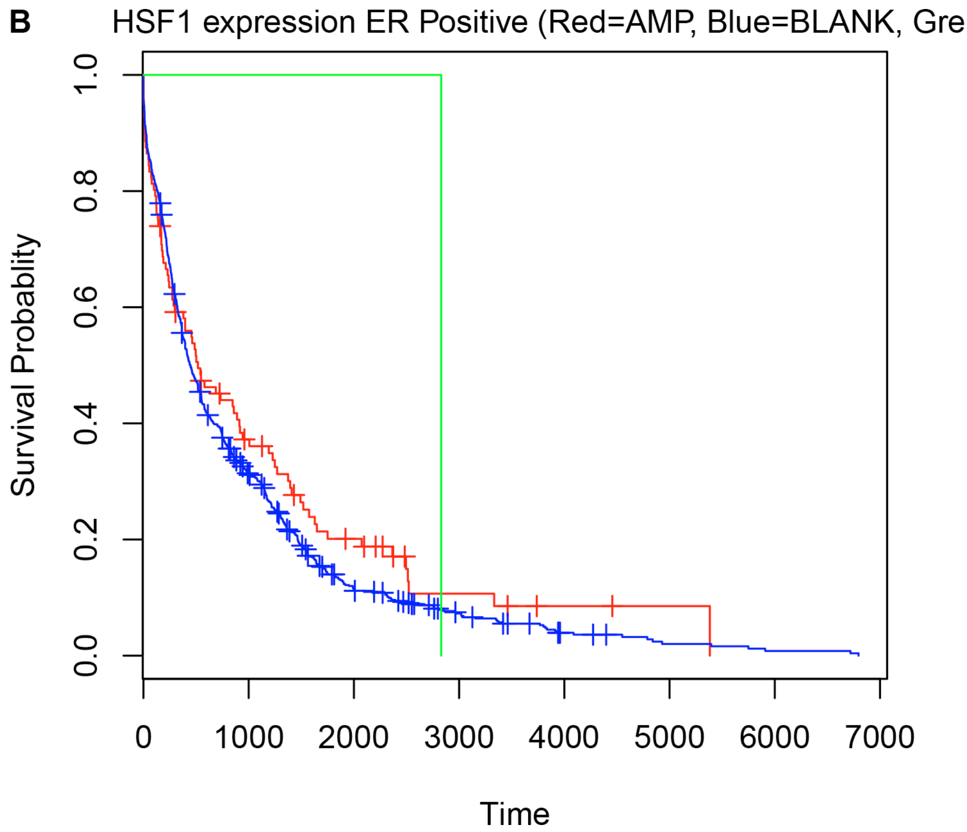

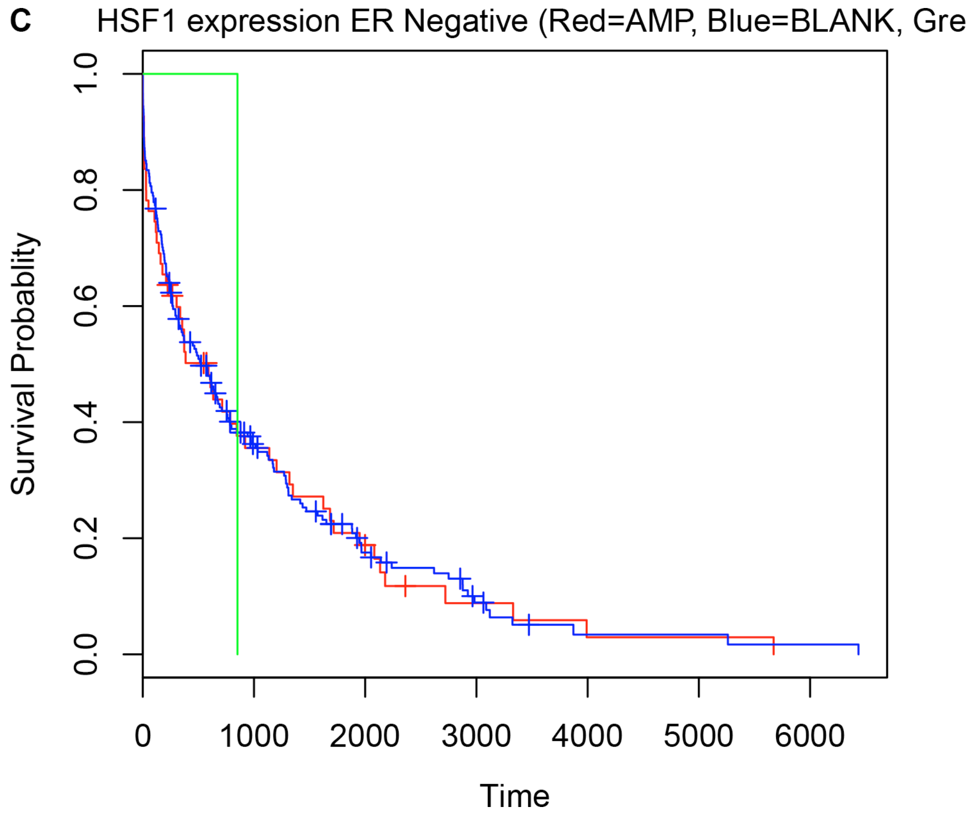

Supplementary Figure S1: Breast cancer overall survival data analyzing the impact of HSF1 amplification in TCGA Breast Cancer dataset.

Supplementary Table S1: Patient age and tumor characteristics of the Oncotype Dx cohort. See Supplementary_Table_S1
